# Retraction Note: Efficacy of using adipose-derived stem cells and PRP on regeneration of 40 -mm long sciatic nerve defect bridged by polyglycolic-polypropylene mesh in canine model

**DOI:** 10.1186/s13287-026-05054-w

**Published:** 2026-05-11

**Authors:** Mona M. Khaled, Asmaa M. Ibrahium, Ahmed I. Abdelgalil, Mohamed A. El-Saied, Aya M. Yassin, Nagy Abouquerin, Hamdy Rizk, Samah H. El-Bably

**Affiliations:** 1 Department of Anatomy & Embryology, Faculty of Veterinary Medicine, Cairo University, Giza, Egypt; 2 Department of Surgery, Anesthesiology and Radiology, Faculty of Veterinary Medicine, Cairo University, Giza, Egypt; 3 Department of Pathology, Faculty of Veterinary of Veterinary Medicine, Cairo University, Giza, Egypt; 4 Department of Biochemistry and Molecular Biology, Faculty of Veterinary Medicine, Cairo University, Giza, Egypt; 5 Department of Physiology, Faculty of medicine, Ain shams University, Cairo, Egypt

**Retraction Note** **: Stem Cell Research & Therapy (2024) 15:212**

10.1186/s13287-024-03796-z

The Editor-in-Chief has retracted this article. After publication, it was found that in Fig. 3, the panel for adipogenic differentiation (in ADSCs) appeared to overlap with Fig. 3, BMSCs panel B (adipogenic differentiation), with rotation, of a previous article by the same author group [1]. The authors submitted a replacement panel which could not be validated to the time of the original experiment, and so could not be used to correct the article. The Editor has lost confidence in the data and conclusions of this article.

Nagy Abouquerin, Samah H. El-Bably, Asmaa M. Ibrahium,, Mona M. Khaled, Ahmed Ismael Abdelgalil and Aya M. Yassin agree with this retraction. Mohamed A. El-Saied and Hamdy Rizk did not respond to correspondence from the publisher regarding this retraction.
